# PTX3 as a key modulator of functional ovarian response in PCOS: evaluation alongside TSG-6 and ITI

**DOI:** 10.1186/s13048-025-01798-w

**Published:** 2025-09-26

**Authors:** Zercan Kalı, Ümran Karabulut, Tuba Memur, Fatma Tanılır Çağıran, Nihal Mavral, Pınar Kırıcı

**Affiliations:** 1 Department of Obstetrics and Gynecology, Private Gözde Hospital, Malatya, Turkey; 2 Department of Obstetrics and Gynecology, Private Acıbadem Hospital, Kayseri, Turkey; 3 Private Obstetrics and Gynecology Clinic, Malatya, Turkey; 4 Private Obstetrics and Gynecology Clinic, Diyarbakır, Turkey; 5 Department of Obstetrics and Gynecology, Malatya Turgut Özal University, Malatya, Turkey

**Keywords:** PTX3, TSG-6, Inter-α-trypsin inhibitor, Follicular fluid, FORT

## Abstract

**Objective:**

To investigate the relationship between follicular fluid pentraxin 3 (PTX-3) levels and ovarian response, embryo quality, and insulin resistance (IR) in patients with polycystic ovary syndrome (PCOS) undergoing IVF/ICSI.

**Methods:**

A total of 130 women were enrolled and categorized into three groups: lean PCOS (*n* = 43), overweight PCOS (*n* = 42), and unexplained infertility (UEI, *n* = 45). Patients with endocrine disorders, chronic inflammatory diseases, or recent hormonal therapy (within 3 months) were excluded. Follicular fluid (FF) and serum PTX-3 levels were measured using ELISA. Subgroup analyses were performed according to BMI and HOMA-IR status. Correlations between FF PTX-3 and clinical, hormonal, and embryological parameters were assessed. ROC curve analysis and multivariate linear regression were used to evaluate the diagnostic and predictive value of FF biomarkers for follicular output rate (FORT).

**Results:**

FF PTX-3 levels were significantly higher in both lean (23.31 ± 1.33 ng/mL) and overweight PCOS patients (12.54 ± 1.05 ng/mL) compared to UEI controls (7.01 ± 0.54 ng/mL; *p* = 0.029). Notably, PTX-3 remained elevated in lean PCOS despite a lower BMI, supporting its role in intrinsic ovarian inflammation. FF PTX-3 showed significant positive correlations with total testosterone (*r* = 0.580), AFC (*r* = 0.598), and oocyte count (*r* = 0.532), but was inversely associated with high-quality embryo number (*r* = − 0.482), 2PN count (*r* = − 0.312), and FORT (*r* = − 0.418). ROC analysis demonstrated moderate diagnostic performance of PTX-3 for predicting suboptimal FORT (AUC = 0.77; cut-off: 20.4 ng/mL). In multivariate analysis, FF PTX-3 (β = − 0.65, *p* = 0.001), TSG-6 (β = − 0.42), and ITI (β = − 0.37) were independent negative predictors of FORT, while AFC was positively associated.

**Conclusion:**

Elevated follicular PTX-3 levels are linked to hyperandrogenism and ovarian reserve in PCOS, but may impair embryo quality and functional follicular response. PTX-3 may serve as a potential biomarker of ovarian inflammation and compromised oocyte competence, independent of BMI or systemic insulin resistance.

## Introduction

PCOS is one of the most common endocrine disorders among women of reproductive age and represents a leading cause of anovulatory infertility [1]. In individuals with PCOS, the response to controlled ovarian stimulation varies considerably, and the relationship between follicle count and oocyte quality directly affects fertilization outcomes.

Given the heterogeneous nature of PCOS—characterized by varying degrees of insulin resistance, hyperandrogenism, and chronic inflammation—it is critical to consider not only static markers of ovarian reserve but also dynamic indicators of ovarian responsiveness. The FORT, a functional parameter that reflects the relationship between ovarian reserve and clinical response, has gained increasing attention for this purpose [2]. Several studies have shown that FORT values above 70% are predictive of optimal oocyte yield and embryo quality [3]. More recently, the concept of “functional ovarian efficiency” has been introduced into the literature, particularly in PCOS protocols stimulated with letrozole, where FORT is utilized as a monitoring parameter [4, 5].

In recent years, research investigating the impact of the inflammatory profile of granulosa cells on oocyte quality in PCOS has grown substantially. This expanding body of evidence indicates that the microinflammatory milieu not only induces localized cellular stress but also plays a pivotal role in oocyte maturation, epigenetic stability, and subsequent embryonic development [6, 7]. Accordingly, proinflammatory cytokines such as IL-6, TNF-α, and IL-18, along with antioxidant defense components like SOD, GPx, and NAD⁺—particularly within the follicular fluid—have become central targets of investigation [8].

Beyond conventional inflammatory markers, recent molecular studies on the immunometabolic characteristics of granulosa cells have led to the identification of novel candidate biomarkers, including miR-34a-5p, TXNIP, and mitochondrial dynamics-related proteins [9, 10]. These developments are expected to further advance in the near future, laying the groundwork for more sensitive and specific diagnostic tools to assess oocyte competence.

In this context, PTX3, a key component of the follicular micromatrix, has also attracted attention. This glycoprotein, a prototypical member of the long pentraxin family, plays essential roles in modulating the immune response and organizing the ECM. PTX3 acts synergistically with hyaluronic acid (HA), TSG-6, and inter-α-trypsin inhibitor ITI to maintain the viscoelastic integrity of the cumulus–oocyte complex [11,12]. In PCOS, dysregulation of PTX3 levels has been reported in both serum and follicular fluid. However, the relationship between these abnormalities and metabolic parameters such as BMI, as well as the FORT, which reflects the quality of follicular response, remains unclear [13, 14].

Despite increasing proteomic studies in the literature focusing on FORT in PCOS, the number of investigations examining the relationship between follicular fluid levels of PTX3, TSG-6, and ITI and their effects on FORT and embryo quality remains very limited. To address this gap, the present study investigated the association between follicular fluid levels of PTX3, TSG-6, and ITI and the FORT ratio in PCOS patients, and further assessed their correlations with clinical parameters such as AMH, AFC, BMI, age, and embryo qualityThis study is among the few pioneering investigations that concurrently assess serum and follicular fluid PTX-3 levels in PCOS patients, in relation to BMI-based subgrouping, follicular response parameters, and reproductive outcomes.

## Material methods

This prospective observational study was conducted at the IVF Unit of Gözde Akademi Hospital between November 2023 and March 2024. A total of 130 women undergoing assisted reproduction were included: 85 patients diagnosed with polycystic ovary syndrome (PCOS) according to the 2003 Rotterdam criteria, and 45 women with unexplained infertility (UEI) served as controls. PCOS diagnosis required the presence of at least two of the following criteria: hyperandrogenism (clinical or biochemical), ovulatory dysfunction (oligo- or amenorrhea), and polycystic ovarian morphology (≥ 12 follicles measuring 2–9 mm in one ovary). Patients with alternative causes of hyperandrogenism (e.g., Cushing’s syndrome, congenital adrenal hyperplasia), thyroid dysfunction, hyperprolactinemia, idiopathic hirsutism, or those who had used hormonal or insulin-sensitizing agents within the past six months were excluded. Endometriosis and tubal pathology were ruled out using clinical and radiologic evaluation, including hysterosalpingography. UEI was defined as the inability to conceive despite 12 months of regular unprotected intercourse, with normal ovulatory function, tubal patency, and semen analysis.

BMI categories were defined according to WHO standards: 18.5–24.9 kg/m² (lean) and 25.0–29.9 kg/m² (overweight). Patients with a BMI ≥ 30.0 kg/m² were excluded to eliminate the potential confounding impact of obesity-related systemic inflammation on follicular microenvironment markers. Hormonal analyses, including LH, FSH, total testosterone, and fasting insulin levels, were conducted using standard immunoassays. In both induced and spontaneous cycles, blood samples for hormonal analysis were collected during the early follicular phase (cycle days 2–4) to ensure consistency across participants. Insulin resistance was calculated using the homeostasis model assessment of insulin resistance (HOMA-IR) formula: HOMA-IR = (fasting insulin [µU/mL] × fasting glucose [mg/dL]) / 405.

FORT was calculated as the ratio of preovulatory follicles to the antral follicle count (AFC).

Preovulatory follicles were defined as those measuring ≥ 17 mm in diameter on the day of ovulation trigger (hCG administration), as assessed by transvaginal ultrasound. AFC was measured on cycle day 3.

All patients underwent controlled ovarian stimulation using a gonadotropin-releasing hormone (GnRH) antagonist protocol. Recombinant FSH (Gonal-F, Merck) was started on day 3 of the menstrual cycle. Cetrorelix acetate (Cetrotide 250 µg) was introduced when the leading follicle reached ≥ 14 mm. Final oocyte maturation was triggered with recombinant hCG (250 µg), and oocyte retrieval was performed 35–36 h. Follicular fluid samples were collected from the largest, mature follicle (17–20 mm in diameter) under transvaginal ultrasound guidance during oocyte retrieval. Only one follicle was aspirated per patient, and the presence of an MII oocyte confirmed that the aspirated follicle was dominant. Samples that were visibly bloody or contaminated were excluded. Serum samples were collected on the day of oocyte retrieval. All samples were centrifuged and stored at − 80 °C until biochemical analysis.

Follicular fluid and serum PTX-3 levels were measured using a commercially available sandwich ELISA kit (Human Pentraxin 3 ELISA Kit, Catalog no. SEA608Hu, Cloud-Clone Corp, Houston, TX, USA). The detection range of the assay was 0.1–30 ng/mL. The minimum detectable concentration (sensitivity) was 0.1 ng/mL. According to the manufacturer’s specifications, intra-assay and inter-assay coefficients of variation (CV) were < 10% and < 12%, respectively. The assay had a detection range of 0.08–20 ng/mL, a sensitivity of 0.051 ng/mL, and intra-/inter-assay coefficients of variation of 10% and 12%, respectively. To evaluate the interactions of PTX-3 with extracellular matrix proteins, immunoprecipitation (IP) assays were performed on follicular fluid and serum samples. After preclearing, 200 µL of each sample was incubated overnight at 4 °C with either anti-PTX-3 monoclonal antibody (R&D Systems, USA) or control IgG. Protein A/G PLUS agarose beads (Santa Cruz Biotechnology, USA) were added to capture the complexes, which were then washed with PBS containing 0.1% Triton X-100 and eluted using SDS sample buffer. Concentrations of TSG-6 and inter-α-trypsin inhibitor (ITI) in follicular fluid were quantitatively measured using commercially available ELISA kits in accordance with the manufacturers’ protocols (Human TSG-6 ELISA Kit, Catalog No: SEA992Hu; Human Inter-alpha-trypsin Inhibitor ELISA Kit, Catalog No: SEA875Hu; Cloud-Clone Corp., Houston, TX, USA).

### Statistical analysis

All statistical analyses were performed using SPSS software version 26.0 (IBM Corp., Armonk, NY, USA). Continuous variables were expressed as mean ± standard deviation (SD) or median (interquartile range), depending on data distribution assessed by the Shapiro–Wilk test. Group comparisons were conducted using one-way ANOVA or the Kruskal–Wallis test, followed by post hoc Bonferroni or Dunn’s tests where appropriate. Categorical variables were analyzed using the chi-square test. Correlations between PTX-3 levels and clinical parameters were assessed with Spearman’s rank correlation. Receiver operating characteristic (ROC) curve analysis was used to evaluate the predictive performance of PTX-3, TSG-6, and ITI for suboptimal ovarian response, with the Youden index used to determine optimal cutoff values. Multivariable linear regression was performed to identify independent predictors of FORT, and p-values below 0.05 were considered statistically significant. HOMA-IR values were compared across the three groups using Welch’s ANOVA due to unequal variances. Post-hoc comparisons were conducted using the Games-Howell test to identify pairwise differences. A *p-value* below 0.05 was considered statistically significant. Paired comparisons between follicular fluid and serum PTX-3 levels within each group were conducted using the Wilcoxon signed-rank test.

## Results

In this IVF cohort, lean PCOS, overweight PCOS, and unexplained infertility (UEI) patients were similar in age but exhibited distinctly different metabolic and follicular profiles (Table 1). Overweight PCOS women had the highest BMI, whereas both PCOS groups showed significantly elevated LH, HOMA-IR, and total testosterone compared with UEI controls. Antral follicle count, preovulatory follicle number, mature oocytes, and both total and high-quality embryo yields peaked in the overweight PCOS group, while the follicular output rate (FORT) was lowest in lean PCOS (64.6 ± 9.2%, *p* = 0.047). Notably, lean PCOS also displayed the highest follicular-fluid levels of PTX-3, TSG-6, and inter-α-trypsin inhibitor (all *p* < 0.05), and required the lowest total gonadotropin dose (*p* < 0.001).

**Table 1:** Clinical and biochemical characteristics of study groups

Parameter	Lean PCOS (*n* = 43)	Overweight PCOS (*n* = 42)	UEI (*n* = 45)
Age (years)	26.86 ± 1.90	25.82 ± 2.16	24.99 ± 2.14
BMI (kg/m²)	23.44 ± 2.31	26.85 ± 2.42	20.70 ± 1.27
AFC	29.33 ± 1.88	31.87 ± 2.60	13.46 ± 0.90
Preovulatory Follicles	19.09 ± 0.99	25.14 ± 1.93	9.67 ± 0.54
FORT (%)	64.63 ± 5.20	73.58 ± 4.01	71.56 ± 6.89
MII oocyte	15.53 ± 0.64	18.89 ± 0.82	8.58 ± 0.52
2PN	9.70 ± 0.35	12.16 ± 0.60	6.94 ± 0.37
Embryo Count	8.22 ± 0.40	10.80 ± 0.40	4.64 ± 0.47
High-Quality Embryo	4.37 ± 0.42	5.22 ± 0.17	2.45 ± 0.22
Serum PTX-3 (ng/mL)	6.67 ± 0.35	6.04 ± 0.40	4.68 ± 0.29
FF PTX-3 (ng/mL)	23.31 ± 1.33	12.54 ± 1.05	7.01 ± 0.54
FF TSG-6 (ng/mL)	18.34 ± 1.37	15.18 ± 0.77	10.90 ± 0.68
FF ITI (µg/mL)	2563.71 ± 254.53	2321.39 ± 175.51	1896.27 ± 103.64
Total Gonadotropin Dose (IU)	2251.05 ± 139.80	2749.28 ± 168.60	3050.43 ± 289.37
LH (mIU/mL)	12.43 ± 1.22 [12.05–12.81]	6.65 ± 0.41 [6.52–6.78]	5.42 ± 0.4 6 [5.28–5.56]
FSH (mIU/mL)	5.85 ± 0.37 [5.74–5.96]	4.85 ± 0.42 [4.72–4.98]	5.18 ± 0.36 [5.07–5.29]
HOMA-IR	3.33 ± 0.25 [3.25–3.41]	3.48 ± 0.24 [3.41–3.55]	2.01 ± 0.19 [1.95–2.07]
Total Testosterone (ng/dL)	43.32 ± 3.21 [42.33–44.31]	49.18 ± 3.52 [48.08–50.28]	33.31 ± 1.89 [32.74–33.88]

Note: Data are presented as mean ± standard deviation. Values in brackets indicate 95% confidence intervals (CI). Post hoc comparisons were corrected for multiple testing using Bonferroni or Games–Howell adjustment, depending on variance homogeneity

In both lean and overweight PCOS patients, FF PTX-3 levels were significantly and positively correlated with total testosterone (*r* = 0.580 and 0.516, respectively), AFC (*r* = 0.598 and 0.650), and total oocyte count (*r* = 0.532 and 0.502), suggesting an association with ovarian reserve and hyperandrogenism. No significant correlations were found with age, BMI, FSH, LH, or HOMA-IR (all *p* > 0.05), indicating a likely localized inflammatory role for PTX-3.

In all groups, FF PTX-3 levels were significantly higher than serum PTX-3 levels (lean PCOS: 23.31 ± 1.33 vs. 6.67 ± 0.35 ng/mL; overweight PCOS: 12.54 ± 1.05 vs. 6.04 ± 0.40 ng/mL; UEI: 7.01 ± 0.54 vs. 4.68 ± 0.29 ng/mL; *p* < 0.001 for all, Wilcoxon signed-rank test), highlighting a pronounced local accumulation in the follicular compartment.

Conversely, FF PTX-3 showed significant inverse correlations with high-quality embryo number (*r* = − 0.482 and − 0.538), 2PN count (*r* = − 0.312 and − 0.384), and FORT (*r* = − 0.418 and − 0.431), supporting its potential negative impact on oocyte developmental competence and follicular efficiency (Table 2).

**Table 2:** Correlation analysis between hormonal/clinical parameters and FF PTX-3 levels in study groups

Variable	r_Lean	p_Lean	r_Overweight	p_Overweight
Age (years)	0.041	0.782	0.030	0.815
BMI (kg/m²)	0.476	0.073	0.355	0.302
Serum PTX-3 (ng/mL)	0.542	0.076	0.327	0.240
LH (mIU/mL)	0.156	0.381	0.141	0.407
FSH (mIU/mL)	-0.102	0.621	0.081	0.677
Total Testosterone (ng/dL)	0.580	0.001*	0.516	0.020*
AFC	0.598	0.045*	0.650	0.000*
Total Oocyte Count	0.532	0.033*	0.502	0.045*
MII Oocyte	0.324	0.347	0.537	0.122
HOMA-IR	0.204	0.228	0.276	0.161
2PN	-0.312	0.049*	-0.384	0.041*
Embryo Count	-0.294	0.052	-0.341	0.046*
High-Quality Embryo	-0.482	0.012*	-0.538	0.028*
FORT (%)	-0.227	0.232	-0.198	0.284

Note: Pearson correlation was used for normally distributed data; Spearman correlation was applied when the distribution was non-normal

**Fig. 1:**
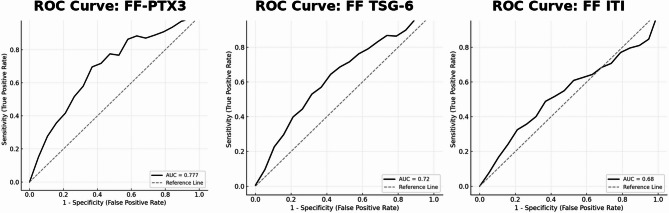
ROC curves for follicular fluid PTX-3, TSG-6, and inter-α-trypsin inhibitor (ITI) in predicting suboptimal ovarian response (FORT < 70%)

Table 3, demonstrates that follicular fluid PTX-3 is the strongest predictor of suboptimal ovarian response (FORT < 70%), achieving an AUC of 0.77 with a Youden-optimized cutoff of 20.4 ng/mL (83% sensitivity, 72% specificity). Inter-α-trypsin inhibitor (ITI) and TSG-6 showed lower discriminative power (AUCs 0.71 and 0.68, respectively). The corresponding ROC curves for all three markers are illustrated in Fig. 1, highlighting PTX-3’s superior curve separation compared with ITI and TSG-6.

**Table 3:** Diagnostic performance of follicular fluid biomarkers in predicting (FORT < 70%)

Biomarker	AUC (95% CI)	*p*-value	Cut-off (ng/mL or µg/mL)	Sensitivity	Specificity
PTX3	0.77 (0.68–0.86)	0.001	20.4	0.83	0.72
Inter-α-trypsin inhibitor	0.71 (0.61–0.80)	0.004	2560.0	0.78	0.67
TSG-6	0.68 (0.58–0.77)	0.013	14.1	0.73	0.63

When the study population was stratified according to follicular fluid PTX-3 levels using a cut-off value of 20.4 ng/mL, significant differences were observed in several clinical and biochemical parameters. Patients with high FF PTX-3 levels (> 20.4 ng/mL; *n* = 58) had significantly lower numbers of high-quality embryos (2.41 ± 0.62 vs. 3.92 ± 0.55; *p* = 0.015) and reduced FORT values (58.12 ± 7.35% vs. 81.25 ± 6.58%; *p* = 0.036) compared to those with low PTX-3 levels (≤ 20.4 ng/mL; *n* = 72). Moreover, FF concentrations of TSG-6 (15.69 ± 1.72 vs. 12.56 ± 2.27 ng/mL; *p* = 0.027) and inter-α-trypsin inhibitor (2412.85 ± 153.99 vs. 2219.02 ± 146.37 µg/mL; *p* = 0.032) were significantly higher in the high PTX-3 group, suggesting an association with increased local inflammatory activity. Although there were no statistically significant differences in AMH levels (*p* = 0.208) or AFC (*p* = 0.318), BMI was significantly lower (21.62 ± 2.42 vs. 25.19 ± 3.59 kg/m²; *p* = 0.043) and age was slightly higher (28.96 ± 2.83 vs. 27.07 ± 3.03 years; *p* = 0.011) in patients with elevated PTX-3 levels. These findings indicate that increased follicular PTX-3 is associated with impaired embryo quality and ovarian response, independent of ovarian reserve markers such as AMH or AFC(Table 4).

**Table 4:** Comparison of clinical and biochemical parameters according to FF PTX3 levels

Variable	High PTX3 (> 20.4 ng/mL) (*n* = 58)	Low PTX3 (≤ 20.4 ng/mL) (*n* = 72)	*p*-value
High-quality embryo (n)	2.41 ± 0.62	3.92 ± 0.55	0.015 *
FORT (%)	58.12 ± 7.35	81.25 ± 6.58	0.036 *
TSG-6 (ng/mL)	15.69 ± 1.72	12.56 ± 2.27	0.027 *
Inter-α-trypsin inhibitor (µg/mL)	2412.85 ± 153.99	2219.02 ± 146.37	0.032 *
AMH (ng/mL)	6.12 ± 1.20	5.79 ± 1.76	0.208
AFC	20.29 ± 5.31	19.43 ± 4.23	0.318
BMI (kg/m²)	21.62 ± 2.42	25.19 ± 3.59	0.043 *
Age (years)	28.96 ± 2.83	27.07 ± 3.03	0.011 *

Note: Data are expressed as mean ± standard deviation. Statistical significance is defined as *p* < 0.05. Welch’s t-test was used for comparisons

In the multivariable linear regression model (R² = 0.52, *p* < 0.001), follicular fluid PTX-3 emerged as the strongest independent predictor of FORT (β = − 0.65, SE = 0.18; *p* = 0.001), with each 1 ng/mL increase in PTX-3 associated with a 0.65%-point decline in FORT. Both FF TSG-6 (β = − 0.42, SE = 0.21; *p* = 0.049) and FF inter-α-trypsin inhibitor (ITI) (β = − 0.37, SE = 0.15; *p* = 0.022) were also significant negative predictors, whereas antral follicle count (AFC) demonstrated a positive association with FORT (β = +0.29, SE = 0.09; *p* = 0.004). In contrast, body mass index (β = − 0.12; *p* = 0.28) and age (β = − 0.09; *p* = 0.34) did not contribute significantly to the model. These findings indicate that local inflammatory mediators—especially PTX-3—rather than systemic factors such as BMI or age, predominantly drive variations in ovarian responsiveness as measured by FORT(Table 5).

**Table 5:** Multiple linear regression analysis of independent predictors of FORT (%)

Variable	β Coefficient	Standard Error	*p*-value	95% Confidence Interval
FF PTX3 (ng/mL)	–0.65	0.18	0.001*	–0.98 − 0.32
FF TSG-6 (ng/mL)	–0.42	0.21	0.049*	–0.84 − 0.01
FF Inter-α-trypsin inhibitor (µg/mL)	–0.37	0.15	0.022*	–0.68–0.06
AFC	0.29	0.09	0.004*	0.10–0.48
(BMI, kg/m²)	–0.12	0.11	0.280	–0.34 − 0.10
Age (years)	–0.09	0.10	0.340	–0.29 − 0.11

Note: Model summary: R² = 0.52, Overall model *p* < 0.001Statistical significance is indicated with an asterisk (*p* < 0.05)

To investigate the impact of insulin resistance on the follicular microenvironment, PCOS patients were stratified into IR− (HOMA-IR ≤ 2.7, *n* = 36) and IR+ (HOMA-IR > 2.7, *n* = 49) subgroups. The IR + group exhibited significantly higher BMI (26.8 ± 2.5 vs. 22.5 ± 2.1 kg/m²; *p* = 0.002) and HOMA-IR levels (3.9 ± 0.5 vs. 2.3 ± 0.4; *p* < 0.001), confirming the expected metabolic distinction.

Follicular fluid PTX-3 concentrations were significantly elevated in the IR + subgroup (18.9 ± 1.7 vs. 14.8 ± 1.5 ng/mL; *p* = 0.047), along with higher levels of FF inter-α-trypsin inhibitor (2456.7 ± 130.6 vs. 2150.4 ± 140.2 µg/mL; *p* = 0.032) and total testosterone (47.6 ± 3.6 vs. 41.2 ± 3.2 ng/dL; *p* = 0.015). Although FF TSG-6 levels were numerically higher in the IR + group, this difference did not reach statistical significance (*p* = 0.063).

Antral follicle count was also significantly greater in IR + patients (31.2 ± 2.8 vs. 27.3 ± 3.1; *p* = 0.041), whereas the follicular output rate (FORT) was statistically comparable between groups (69.5 ± 6.1 vs. 67.2 ± 6.5; *p* = 0.287).

These findings suggest that insulin resistance in PCOS is associated with a more inflamed follicular environment and increased androgenic and follicular activity. However, it does not appear to alter FORT significantly(Table 6).

**Table 6:** Subgroup comparison of PTX-3 and related parameters in ir − and IR + PCOS

Parameter	IR− (HOMA-IR ≤ 2.7, *n* = 36)	IR+ (HOMA-IR > 2.7, *n* = 49)	*p*-value
BMI (kg/m²)	22.5 ± 2.1	26.8 ± 2.5	0.002*
HOMA-IR	2.3 ± 0.4	3.9 ± 0.5	< 0.001*
FF PTX-3 (ng/mL)	14.8 ± 1.5	18.9 ± 1.7	0.047*
FF TSG-6 (ng/mL)	12.1 ± 1.3	14.5 ± 1.4	0.063
FF ITI (µg/mL)	2150.4 ± 140.2	2456.7 ± 130.6	0.032*
Total Testosterone (ng/dL)	41.2 ± 3.2	47.6 ± 3.6	0.015*
AFC	27.3 ± 3.1	31.2 ± 2.8	0.041*
FORT (%)	67.2 ± 6.5	69.5 ± 6.1	0.287

Note: Data are presented as mean ± standard deviation. Statistical comparisons were performed using Welch’s t-test. A p-value < 0.05 was considered statistically significant

## Discussion

PCOS is a complex endocrine disorder characterized by chronic low-grade inflammation within the ovarian microenvironment. Elevated inflammatory responses can disrupt granulosa cell paracrine signaling, thereby impairing oocyte maturation and embryo development (10). In this context, inflammation-related biomarkers such as PTX-3 have emerged as informative indicators of both immunological activity and extracellular matrix dynamics in the follicular milieu of PCOS [15–17].

PTX-3 is a multifunctional protein with established roles in follicular maturation and fertilization. It is synthesized by cumulus oophorus cells in response to LH/hCG stimulation and contributes to the formation of a hyaluronan-rich extracellular matrix. In murine models, deletion of the PTX-3 gene leads to abnormal cumulus expansion and infertility(18,19). Baranova et al. (2022) demonstrated that PTX-3 forms stable cross-linking structures by interacting with TSG-6 and the heavy chains of inter-α-trypsin inhibitors (IαI), stabilizing the HA-based matrix essential for fertilization [20]. Thus, PTX-3 functions not only as a structural component but also as a key organizer that maintains the integrity, elasticity, and signaling competence of the cumulus-oocyte complex [21–23].

However, excessive accumulation of PTX-3 beyond physiological levels may compromise this delicate balance. Overabundance of PTX-3 could lead to excessive cross-linking within the HA–TSG-6–IαI complex, resulting in reduced matrix elasticity and impaired oocyte maturation or sperm penetration. Additionally, PTX-3’s interaction with ligands such as FGF-2 may contribute to aberrant angiogenesis and extracellular remodeling [24, 25]. The complex mesh formed by PTX-3 through its interaction with hyaluronic acid and the heavy chains of inter-α-inhibitor proteins not only facilitates the mechanical entrapment of the oocyte within the fallopian tube but also modulates the inflammatory response within the follicular microenvironment.Thus, elevated PTX-3 levels under pro-inflammatory conditions may reflect a compensatory mechanism aimed at protecting the oocyte from cellular stress [26].

PTX-3 has been identified as a critical molecule that regulates both structural matrix organization and the inflammatory response within the ovarian microenvironment. Considering its dual role, the primary aim of our study was to evaluate the impact of PTX-3 levels on functional ovarian response and reproductive outcomes in PCOS. In this context, follicular fluid samples were collected in a standardized manner from preovulatory follicles measuring 17–20 mm in diameter, each confirmed to contain an MII oocyte. This ensured biological consistency across participants and minimized variability due to follicular maturation status. In contrast, many previous studies collected FF samples at varying follicular stages or did not specify the maturity of the aspirated follicles, which may have contributed to inconsistencies in PTX-3 levels across reports [19, 20]. By implementing a strict sampling protocol, our study aimed to reduce methodological heterogeneity and improve the interpretability of PTX-3 as a follicular biomarker in the context of PCOS.

Additionally, individuals with BMI ≥ 30 kg/m² were deliberately excluded to minimize the confounding effect of obesity-related systemic inflammation on local follicular markers. This approach allowed us to better isolate PTX-3 elevation attributable to intrinsic ovarian inflammation in PCOS rather than to adiposity alone.

Consistent with our findings, follicular fluid PTX-3 levels were significantly elevated in both lean (23.31 ± 1.33 ng/mL) and overweight (12.54 ± 1.05 ng/mL) PCOS patients compared to normo-ovulatory UEI controls (7.01 ± 0.54 ng/mL; *p* = 0.029). Notably, PTX-3 levels remained markedly elevated in the lean PCOS group despite having a lower BMI (23.44 ± 2.31 vs. 26.85 ± 2.42 kg/m²), suggesting that increased PTX-3 reflects intrinsic ovarian inflammation rather than being solely attributable to adiposity.

This study demonstrated that FF PTX-3 levels were positively associated with total testosterone (*r* = 0.580), AFC (*r* = 0.598), and oocyte count (*r* = 0.532), but inversely correlated with high-quality embryo number (*r* = − 0.482), 2PN count (*r* = − 0.312), and FORT (*r* = − 0.418). These findings suggest that elevated PTX-3 may reflect a localized inflammatory response associated with hyperandrogenism and increased follicular activity, while potentially impairing oocyte competence and embryo development.

Our results are consistent with prior studies indicating that high follicular PTX-3 concentrations may disrupt the cumulus-oocyte matrix and negatively influence fertilization and embryo quality [9, 12]. Furthermore, the inverse relationship between PTX-3 and FORT aligns with its proposed inhibitory role in functional follicular recruitment. This was further supported by multivariable regression analysis, where FF PTX-3 emerged as the strongest independent predictor of FORT (β = − 0.65, *p* = 0.001). Given these dual associations—positive with follicular density and negative with reproductive outcomes—PTX-3 may serve as a potential biomarker of dysregulated ovarian microenvironment in PCOS.

ROC analysis demonstrated that follicular PTX-3 had moderate predictive performance for FORT (AUC: 0.77; sensitivity: 83%; specificity: 72%). In comparison, ITI (AUC: 0.71) and TSG-6 (AUC: 0.68) had lower diagnostic performance, implying that PTX-3 may be more directly involved in modulating functional ovarian response. This is in line with reports that PTX-3 governs the activation and localization of these matrix-associated proteins [27–29].

Previous studies investigating FF PTX-3 concentrations have mostly focused on general IVF populations rather than specific PCOS subtypes. For instance, Baranova et al. (2015) demonstrated elevated PTX-3 levels in FF samples of patients with poor ovarian response, while not differentiating between lean and overweight PCOS phenotypes [20]. In contrast, our study specifically stratified PCOS patients based on BMI and revealed a distinct pattern, where elevated FF PTX-3 levels were more prominent in lean PCOS. Moreover, while most prior reports assessed FF samples collected after final oocyte maturation, we standardized sampling in the preovulatory window (follicles 17–20 mm, MII oocytes), which may reduce variability. These methodological distinctions likely contribute to the unique associations we observed between PTX-3 and FORT or embryo quality.

Patients with PTX-3 levels above 20.4 ng/mL exhibited significantly reduced FORT values (58.1 ± 7.4% vs. 81.2 ± 6.6%; *p* = 0.036), supporting the hypothesis that PTX-3 may impair follicular recruitment or responsiveness beyond its role in inflammation. Interestingly, while PTX-3 was positively correlated with LH, AFC, and total oocyte count in both lean and overweight PCOS subgroups, the inverse correlation with FORT suggests that higher recruitment does not translate into better functional efficiency. This underscores the complex interplay between inflammation and follicular dynamics in PCOS.

Group comparisons revealed that high PTX-3 levels (> 20.4 ng/mL) were associated with lower FORT (58.1% vs. 81.2%) and fewer high-quality embryos (2.41 vs. 3.92; *p* < 0.05). Additionally, TSG-6 and ITI were significantly elevated in the high PTX-3 group, whereas AMH and AFC levels were comparable, reinforcing the idea that inflammatory remodeling—rather than reserve status—contributes to impaired outcomes. BMI was lower and age slightly higher in the high PTX-3 group, indicating that PTX-3’s role is not merely a reflection of patient characteristics.

Multivariate regression confirmed follicular PTX-3 (β = − 0.65; *p* = 0.001), TSG-6 (β = − 0.42; *p* = 0.049), and ITI (β = − 0.37; *p* = 0.022) as independent negative predictors of FORT. In contrast, AFC had a positive effect (β = 0.29; *p* = 0.004), while BMI and age were not significant contributors (*p* > 0.2). This highlights that the functional outcome of ovarian stimulation is governed more by inflammatory activity within the follicular microenvironment than by reserve or demographic factors.

Our HOMA-IR values further illustrate this complexity: 3.33 ± 0.25 in the lean PCOS group, 3.48 ± 0.24 in the overweight PCOS group, and 2.01 ± 0.19 in the unexplained infertility (UEI) control group (*p* = 0.0089). These findings support the notion that insulin resistance in PCOS is not solely dependent on adiposity but is instead an intrinsic feature of the syndrome’s metabolic phenotype. Interestingly, no significant correlation was found between follicular fluid PTX-3 levels and HOMA-IR (lean PCOS: *r* = 0.204; overweight PCOS: *r* = 0.276; *p* > 0.05). This result indicates that PTX-3 levels do not exhibit a linear association with individual HOMA-IR variability.

However, in the subgroup analysis based on insulin resistance status, follicular fluid PTX-3 levels were significantly higher in IR + PCOS patients. This finding is consistent with previous literature suggesting that PTX-3 reflects chronic low-grade inflammation associated with insulin resistance and metabolic dysregulation [33]. Although earlier studies such as Tosi et al. (2014) did not find a significant correlation between PTX-3 and HOMA-IR, our direct group comparison suggests that PTX-3 elevation may still occur in the presence of insulin resistance, possibly linked to increased androgen activity and an inflammatory ovarian microenvironment. Moreover, elevated follicular fluid ITI and total testosterone levels in IR + patients further support the presence of an enhanced inflammatory and androgenic response. Nonetheless, FORT values were similar between IR subgroups, suggesting that follicular utilization efficiency may be independent of insulin resistance [30–32].

Although FF PTX-3 levels did not show a significant linear correlation with HOMA-IR when assessed as a continuous variable, subgroup analysis based on a HOMA-IR threshold (> 2.7) revealed that FF PTX-3 concentrations were significantly higher in insulin-resistant (IR+) PCOS patients. This suggests that while the association may not be strongly linear, PTX-3 levels tend to rise above a certain threshold, possibly reflecting a threshold-dependent inflammatory response.

In the same study, Tosi et al. (2014) reported significantly lower plasma PTX-3 levels in women with PCOS compared to healthy controls. However, that study provided limited insight into the extent to which PTX-3 reflects local (ovarian) inflammation. In contrast, our study utilized follicular fluid sampling, which more directly captures the biochemical changes within the ovarian microenvironment. In this context, the significant PTX-3 elevation observed in IR + individuals may better reflect the link between metabolic dysfunction and local inflammation. Our study also differs in methodology and sampling: individuals with a BMI ≥ 30 kg/m² were excluded to minimize the confounding effect of obesity-related systemic inflammation. This allowed us to evaluate whether PTX-3 elevation is associated with intrinsic PCOS-related mechanisms rather than adiposity. Additionally, PCOS patients were not analyzed as a single group; instead, subgroup analyses were performed based on body composition (lean vs. overweight) and metabolic phenotype (IR + vs. IR−). These stratifications revealed distinct PTX-3 dynamics across PCOS subtypes and may help explain the discrepancies with the findings of Tosi et al. [30].

### Strengths and limitations

A major strength of this study lies in its dual assessment of PTX-3 in serum and follicular fluid, combined with functional outcome measures such as FORT. Additionally, co-immunoprecipitation assays provided mechanistic insight into PTX-3’s interactions with TSG-6 and ITI.

However, the study has limitations. It was single-centered with a relatively small sample size and did not assess long-term outcomes such as pregnancy or live birth. PTX-3 was measured only at a single time point (oocyte retrieval), precluding dynamic profiling across the stimulation cycle. Future studies should evaluate PTX-3 at multiple phases (e.g., pre-stimulation, hCG trigger, retrieval) to better capture its temporal behavior. Moreover, in vitro models that modulate PTX-3 expression in cumulus cells could further elucidate its role in matrix remodeling and oocyte competence. Finally, prospective studies evaluating PTX-3 as a predictive marker or therapeutic target in PCOS-related infertility are warranted.

## Conclusion

Elevated follicular fluid PTX-3 levels were significantly associated with reduced FORT and poorer embryo quality. ROC analysis confirmed its diagnostic value in predicting suboptimal ovarian response. These findings suggest that PTX-3 may serve as a clinically relevant biomarker in PCOS-related infertility and play a key role in the regulation of inflammation within the ovarian microenvironment.

## Data Availability

The datasets generated and/or analyzed during the current study are available from the corresponding author upon reasonable request.
